# Does Exposure to General Anesthesia Increase Risk of ADHD for Children Before Age of Three?

**DOI:** 10.3389/fpsyt.2021.717093

**Published:** 2021-11-24

**Authors:** Junjie Song, Huifang Li, Ying Wang, Chenguang Niu

**Affiliations:** ^1^Department of Anesthesiology, The First Affiliated Hospital of Henan University, Kaifeng, China; ^2^Department of Medical Hospital, Henan University, Henan Medical Hospital, Henan University, Kaifeng, China; ^3^The Key Laboratory of Clinical Resources Translation, Henan University, Kaifeng, China

**Keywords:** general anesthesia, ADHD, children, meta-analysis, single, multiple

## Abstract

**Objective:** The objective of the study was to evaluate the relationship between frequency of exposure to general anesthesia before the age of 3 and subsequent risk of diagnosis for attention-deficit hyperactivity disorder (ADHD).

**Method:** We searched PubMed, Embase, Web of Science, and Cochrane Library database for eligible inclusion in the meta-analysis. The indicated outcomes were extracted from the included studies, and the combined effects were calculated using the RevMan software 5.3.

**Results:** Compared with no exposure to general anesthesia, single exposure to general anesthesia did not increase the risk of ADHD for children before the age of 3 [hazard ratio (HR): 1.14, 95%; confidence intervals (CI): 0.97–1.35; *p* = 0.11; *I*^2^ = 0%], while multiple exposures to general anesthesia did increase the risk of ADHD (HR: 1.83; 95% CIs: 1.00–3.32; *p* = 0.05; *I*^2^ = 81%).

**Conclusion:** Multiple, but not single, exposures to general anesthesia in children before age of 3 increased the risk of ADHD.

## Introduction

Attention-deficit hyperactivity disorder (ADHD) is a neuropsychological disorder, which is characterized by hyperactivity, impulsivity, inattention, lack of vigilance, and inability to adapt to the rapid changes (1). Children with ADHD typically exhibit deficits in vigilance, verbal learning, working memory, and measures of executive function (2). According to the survey, the prevalence of ADHD in children and adolescents has reached 7.2% worldwide (3). The causes of ADHD are not known well. Although ADHD is a heritable disorder, the gene–environment interaction may be important for its clinical symptoms (4). Environmental factors, such as maternal smoking, prenatal alcohol exposure, viral infections, nutritional deficiencies, low parental education level, perinatal stress, and others may be associated with ADHD (5).

Anesthesia is a state of unconsciousness and painlessness required for unpleasant invasive therapeutic or diagnostic procedures, and general anesthesia is a common anesthesia method for inhibiting the function of the central nervous system. Exposure to a neurotoxic agent, such as anesthetics, could be one of the environmental factors of ADHD. Currently, many studies have reported a relationship between anesthetic exposure and neurodevelopmental outcomes (including language and learning abilities, cognition, behavioral development, and academic performance) in children (6, 7), which implies that the exposure to general anesthesia may increase the risk of ADHD.

To our knowledge, there is no consensus on whether single or multiple exposures to general anesthesia increases subsequent risk of ADHD for young children before the age of 3. Therefore, we performed this meta-analysis to elucidate the effect of single or multiple exposure(s) to general anesthesia on subsequent diagnosis of ADHD for children before the age of 3, and then provide guidance for pediatric practitioners taking care of young children undergoing surgical procedures with general anesthesia at an early age.

## Methods

### Data Sources and Search Strategy

We conducted the meta-analysis according to MOOSE (Meta-Analysis Of Observational Studies in Epidemiology: a proposal for reporting) guidelines. We have searched the following databases (inception to April 2021): PubMed, Embase, Web of Science, and the Cochrane Register of Controlled Trials. The search strategy was specific for each database by using the following search terms: [(including the keyword “anesthesia” or a MeSH search using “General anesthetics”) and (including keyword “pediatric” or “child” or “children” or “newborns” or “neonate” or “young”) and (including a MeSH search “attention-deficit/hyperactivity disorder”)]. A manual search was also performed for relevant references from the selected articles and published reviews.

### Study Selection

Related studies were included on the basis of the following criteria: (1) focused on the relationship between frequency of exposure to procedures requiring general anesthesia for children before the age of 3 and the subsequent risk of ADHD; (2) with sufficient available data to estimate the hazard ratio (HR) with 95% confidence intervals (CI). Only those studies published in the English language were included; we did not define the minimum number of cases in studies to be included for meta-analysis.

### Data Extraction

We obtained data about the general characteristics (study design, country) and patient characteristics (birth, the age of exposure to general anesthesia) from studies. Times of exposure to general anesthesia and the risk of ADHD for children was also extracted as a major outcome (Table 1). All articles were examined independently for eligibility by two reviewers (SJJ, LHF). Disagreements were resolved by consultation with a third reviewer (NCG).

**Table 1:** Summary of included studies.

**References**	**Design type**	**Country**	**Birth year**	**Age of GA exposure**	**Number of GA exposure**
Sprung et al. (11)	Retrospective cohort study	Rochester	1976–1982	Before age 2	0 (*n* = 4,156) 1 (*n* = 226) ≥2 (*n* = 43)
Ko et al. (10)	Retrospective matched-cohort study	Taiwan	2001–2005	Before age 3	0 (*n* = 13,172) 1 (*n* = 2,019) ≥2 (*n* = 1,274)
Hu et al. (9)	Retrospective cohort study	Olmsted County, Minnesota	1996–2000	Before age 3	0 (*n* = 463) 1 (*n* = 457) ≥2 (*n* = 116)
Tsai et al. (12)	Retrospective cohort study	Taiwan	1997–1999	Before age 3	0 (*n* = 3438) 1 (*n* = 804) ≥2 (*n* = 342)

*GA, general anesthesia*.

### Statistical Analysis

We performed statistical analyses with Review Manager (RevMan 5.3, Cochrane Collaboration, Nordic Cochrane Center, Copenhagen, Denmark). For each study, HR was retrieved to estimate the association between the times of anesthesia exposure and the risk of ADHD. The combined HRs and their 95% CI were considered as the effect sizes for calculating the merged results. Using Cochrane-based *I*^2^-test (8), heterogeneity test was performed for the studies. When there was a significant heterogeneity among the studies (*I*^2^ > 50%), the random effects model was applied. On the contrary, the fixed effects model was used when homogeneous outcomes were obtained (*I*^2^ < 50%). Results were considered as statistically significant for *p* < 0.05.

## Results

### Description of Included Studies

**Figure 1:**
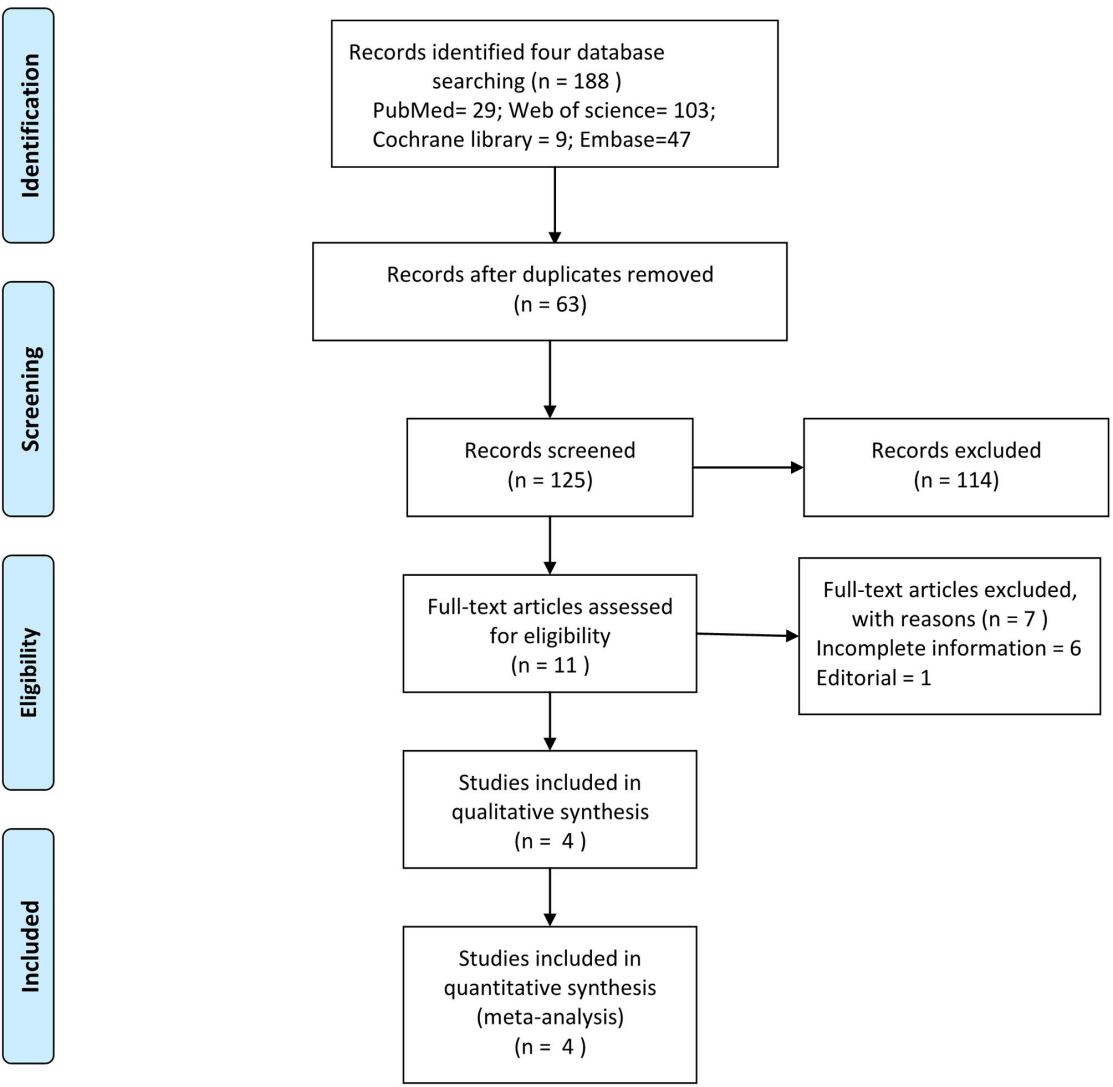
Flow diagram for the study identified and included into this meta-analysis.

The flow diagram of study selection is shown in Figure 1. By the search strategy, 188 records were obtained, including 29 articles in PubMed, 103 articles in Web of Science, 47 articles in Embase, and 9 articles in the Cochrane Library database (from 1950 to April 2021). After removing 63 repeated records, 125 records remained. Then 114 records were excluded after browsing the title and reading the abstract. Furthermore, 11 records were screened out following the full-text reading. After reviewing the full-text of all possibly eligible articles, four eligible studies were selected for the present meta-analysis (9–12). A detailed flow for the study inclusion is shown in Figure 1.

A total of 26,510 children were included, among which, 3,506 children had experienced single anesthesia, and 1,775 children had experienced multiple exposure to anesthesia due to surgery procedures before the age of 3. Of the four included studies, one was conducted in Olmsted County, Minnesota, USA (9), two in Taiwan, China (10, 12), and one in Rochester, MN, USA (11). Three studies reported the time length of the total duration of anesthesia exposure (9, 11). One study excluded children with learning disabilities (11). One study relied on the documentation within medical and school records of ADHD diagnoses and questionnaires to identify the ADHD (9), and the other two papers utilized ICD-9-CM (10, 12) and DSM-IV (11).

### Outcome Analysis

All articles reported the adjusted HR for the association between the times of exposure to general anesthesia and the risk of ADHD. Data in the study of Hu were adjusted for sex, birth weight, gestational age, mother's education, and socioeconomic status (9). Data in the study of Ko were adjusted for place of residence, parental occupation, perinatal conditions, and congenital anomalies (10). Data in the study of Spruing were adjusted for sex, birth weight, and gestational age (11). Data in the study of Tsai were adjusted for gestation age, sex, living area, parental economic status, parental occupation, and comorbid health condition (12).

**Figure 2:**
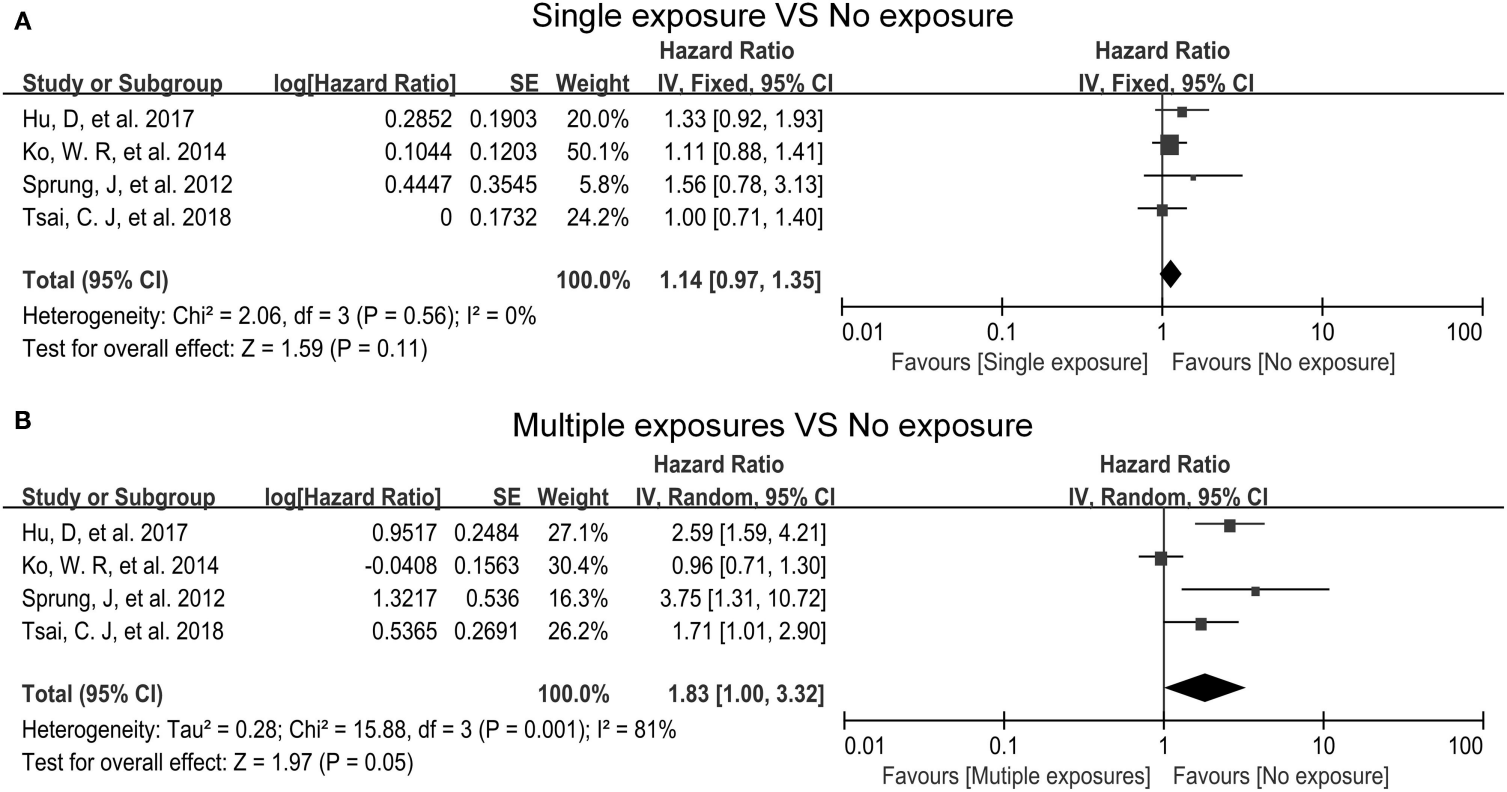
Forest plots for association between exposure times and risk of attention-deficit hyperactivity disorder (ADHD). **(A)** Single exposure do not increase the risk of ADHD compared with no exposure. **(B)** Multiple exposures increase the risk of ADHD compared with no exposure.

Because there was no significant heterogeneity (*I*^2^ = 0.0%), a fixed-effects model was used to combine adjusted HRs of single exposure vs. no exposure. As shown in Figure 2A, single exposure was not significantly associated with an increased risk of ADHD [HR = 1.14, 95% = (0.97–1.35), *p* = 0.11].

Because there was significant heterogeneity (*I*^2^ = 81%), a random-effects model was used to combine adjusted HRs of multiple exposures vs. no exposure. As shown in Figure 2B, multiple exposures did associate with an increased risk of ADHD [HR = 1.83, 95% CI = (1.00–3.32), *P* = 0.05].

## Discussion

To our knowledge, this is the first meta-analysis to reveal the relationship between the frequency of exposure to general anesthesia and the risk of ADHD in children before age of 3. The major finding of our meta-analysis was that single exposure to general anesthesia might not be responsible for ADHD in children before the age of 3, but multiple exposures to general anesthesia could increase the risk of ADHD in children before the age of 3 (Figure 2).

Though this meta-analysis shows that there is no significant difference of subsequent ADHD risk between none and single anesthesia, some proximate studies display an inconsistent statistical result. A matched study showed that children who undergo single minor surgery requiring anesthesia under the age of 5 had a small significantly increased risk of ADHD diagnoses (13). Another twin study found that ADHD was slightly associated with single anesthesia under 0–12 years (14). Superficially, it seems that the above researches challenge the result from this meta-analysis, but in truth, their data are in accordance with ours, because their HR values are similar with ours. Besides, the study design and population age in the above researches are different from the included studies, so there is conflict even if the risks of ADHD caused by anesthesia were varied.

Compared with no general anesthesia, multiple general anesthesia increased the risk of ADHD, and the HR is up to 1.83 with a statistical significance. We think one of the possible reasons for this result is the total duration of anesthesia exposure and the total usage amount of anesthesia agent. Children receiving multiple general anesthesia may suffer a long total duration of exposure and thereby take more anesthetic. Relevant studies have revealed that exposing to general anesthesia agents would duration-dependently and dosage-dependently increase the risk of neurodevelopmental deficit (15, 16). In addition, repeated use of inhalation anesthetics, such as sevoflurane, in multiple general anesthesia, may be one reason for increasing the risk of ADHD. Several experiments demonstrated that single exposure to sevoflurane could not cause significant neuronal apoptosis or long-term neurocognitive impairment (17, 18), but when sevoflurane was used again, the mitochondrial apoptotic pathways would be activated, causing significant apoptosis of hippocampal neurons that is vital for learning and memory (17, 18). Impaired hippocampal function may then increase the risk of ADHD.

In this meta-analysis, we have chosen the children who were exposed before the age of 3 years as the study population. In general speaking, the age of 3 years was used frequently as a definition of early life (19, 20). The period of synaptogenesis has been considered to extend through the age of 3 years in humans (21), and the period from birth to the age of 2–4 years is suspected to be the period of brain vulnerability. Besides, we have noticed a warning of the U.S. Food and Drug Administration (FDA). The warning informed that repeated or lengthy use of general anesthetics during surgeries or procedures in children younger than 3 years may affect the development of the brains of children (22).

Significant heterogeneity was detected among HRs of multiple exposures to general anesthesia vs. no exposure in this meta-analysis. By sensitivity analysis, we found that heterogeneity disappeared when the study of Ko was excluded (10). In the study of Ko, the sample size was relatively big, with a total of 16,465 samples; therefore, a smaller standard error of HR was exhibited. In addition, the study is a matched cohort, and its HR was not adjusted for gender, age, and weight because it was not necessary; however, the other three studies are independent cohorts, and their HRs were adjusted for the above factors. So we speculate all these may cause its HR and standard error to be different from the others.

The major limitation of this meta-analysis is that all studies included were observational retrospective studies due to the lack of randomized controlled study. The observational cohort studies are inherently biased on selection and confounding, though these do provide real-world results. The other limitation is that children who require anesthesia might differ in healthy status from those who do not. Beyond that, children receiving anesthesia also experienced surgery or other procedures at the same time, and it is possible that the factors associated with the surgery or procedure could be the risk factor of ADHD. Consequently, we were not able to distinguish the effect of anesthesia by itself on ADHD from the potential effect of the surgery or procedure.

In summary, multiple exposures, but not single, to general anesthesia before the age of 3 increased later risk of ADHD.

## Data Availability Statement

The original contributions presented in the study are included in the article/supplementary material, further inquiries can be directed to the corresponding author/s.

## Author Contributions

JS conceptualized and designed the study, drafted the initial manuscript, and reviewed and revised the manuscript. HL carried out the initial analyses, and reviewed and revised the manuscript. YW and CN conceptualized and designed the study, coordinated and supervised the data collection, and critically reviewed the manuscript for important intellectual content. All authors approved the final manuscript as submitted and agreed to be accountable for all aspects of the work.

## Funding

This study was supported by the Henan Provincial Science and Technology Department (No. 142300410129), Henan Provincial Education Department (No. 19A320021), and National Natural Science Foundation of China (Nos. 81800395 and 81600940).

## Conflict of Interest

The authors declare that the research was conducted in the absence of any commercial or financial relationships that could be construed as a potential conflict of interest.

## Publisher's Note

All claims expressed in this article are solely those of the authors and do not necessarily represent those of their affiliated organizations, or those of the publisher, the editors and the reviewers. Any product that may be evaluated in this article, or claim that may be made by its manufacturer, is not guaranteed or endorsed by the publisher.

## Abbreviations

ADHD: attention-deficit hyperactivity disorder
HR: hazard ratio
CI: confidence intervals.

## References

[1] Zheng J Gao Y Xu X Kang K Liu H Wang H . Correlation of bispectral index and Richmond agitation sedation scale for evaluating sedation depth: a retrospective study. J Thorac Dis. (2018) 10:190–5. 10.21037/jtd.2017.11.129 29600048 PMC5863203

[2] Seidman LJ. Neuropsychological functioning in people with ADHD across the lifespan. Clin Psychol Rev. (2006) 26:466–85. 10.1016/j.cpr.2006.01.004 16473440

[3] Thomas R Sanders S Doust J Beller E Glasziou P. Prevalence of attention-deficit/hyperactivity disorder: a systematic review and meta-analysis. Pediatrics. (2015) 135:e994–1001. 10.1542/peds.2014-3482 25733754

[4] Thapar A Langley K Asherson P Gill M. Gene-environment interplay in attention-deficit hyperactivity disorder and the importance of a developmental perspective. Br J Psychiatry. (2007) 190:1–3. 10.1192/bjp.bp.106.027003 17197648

[5] Spencer T Biederman J Wilens T Guite J Harding M. ADHD and thyroid abnormalities: a research note. J Child Psychol Psychiatry. (1995) 36:879–85. 10.1111/j.1469-7610.1995.tb01335.x 7559851

[6] Wang X Xu Z Miao CH. Current clinical evidence on the effect of general anesthesia on neurodevelopment in children: an updated systematic review with meta-regression. PLoS ONE. (2014) 9:e85760. 10.1371/journal.pone.0085760 24465688 PMC3896404

[7] Zhang H Du L Du Z Jiang H Han D Li Q. Association between childhood exposure to single general anesthesia and neurodevelopment: a systematic review and meta-analysis of cohort study. J Anesth. (2015) 29:749–57. 10.1007/s00540-015-2030-z 26002228

[8] Higgins JP Thompson SG Deeks JJ Altman DG. Measuring inconsistency in meta-analyses. BMJ. (2003) 327:557–60. 10.1136/bmj.327.7414.557 12958120 PMC192859

[9] Hu D Flick RP Zaccariello MJ Colligan RC Katusic SK Schroeder DR . Association between exposure of young children to procedures requiring general anesthesia and learning and behavioral outcomes in a population-based birth cohort. Anesthesiology. (2017) 127:227–40. 10.1097/ALN.0000000000001735 28609302 PMC5515677

[10] Ko WR Liaw YP Huang JY Zhao DH Chang HC Ko PC . Exposure to general anesthesia in early life and the risk of attention deficit/hyperactivity disorder development: a nationwide, retrospective matched-cohort study. Paediatr Anaesth. (2014) 24:741–8. 10.1111/pan.12371 24612161

[11] Sprung J Flick RP Katusic SK Colligan RC Barbaresi WJ Bojanić K . Attention-deficit/hyperactivity disorder after early exposure to procedures requiring general anesthesia. Mayo Clin Proc. (2012) 87:120–9. 10.1016/j.mayocp.2011.11.008 22305025 PMC3538403

[12] Tsai CJ Lee CT Liang SH Tsai PJ Chen VC Gossop M. Risk of ADHD after multiple exposures to general anesthesia: a nationwide retrospective cohort study. J Atten Disord. (2018) 22:229–39. 10.1177/1087054715587094 26023173

[13] Ing C Sun M Olfson M DiMaggio CJ Sun LS Wall MM . Age at exposure to surgery and anesthesia in children and association with mental disorder diagnosis. Anesth Analg. (2017) 125:1988–98. 10.1213/ANE.0000000000002423 28857799 PMC5856466

[14] Castellheim A Lundström S Molin M Kuja-Halkola R Gillberg C Gillberg C. The role of general anesthesia on traits of neurodevelopmental disorders in a Swedish cohort of twins. J Child Psychol Psychiatry. (2018) 59:966–72. 10.1111/jcpp.12885 29465765

[15] Yon JH Daniel-Johnson J Carter LB Jevtovic-Todorovic V. Anesthesia induces neuronal cell death in the developing rat brain via the intrinsic and extrinsic apoptotic pathways. Neuroscience. (2005)135:815–27. 10.1016/j.neuroscience.2005.03.064 16154281

[16] Satomoto M Satoh Y Terui K Miyao H Takishima K Ito M . Neonatal exposure to sevoflurane induces abnormal social behaviors and deficits in fear conditioning in mice. Anesthesiology. (2009) 110:628–37. 10.1097/ALN.0b013e3181974fa2 19212262

[17] Huang H Liu CM Sun J Jin WJ Wu YQ Chen J. Repeated 2% sevoflurane administration in 7- and 60-day-old rats: neurotoxicity and neurocognitive dysfunction. Anaesthesist. (2017) 66:850–7. 10.1007/s00101-017-0359-4 28914327

[18] Amrock LG Starner ML Murphy KL Baxter MG. Long-term effects of single or multiple neonatal sevoflurane exposures on rat hippocampal ultrastructure. Anesthesiology. (2015) 122:87–95. 10.1097/ALN.0000000000000477 25289484

[19] DiMaggio C Sun LS Li G. Early childhood exposure to anesthesia and risk of developmental and behavioral disorders in a sibling birth cohort. Anesth Analg. (2011) 113:1143–51. 10.1213/ANE.0b013e3182147f42 21415431 PMC3164160

[20] Ing C DiMaggio C Whitehouse A Hegarty MK Brady J von Ungern-Sternberg BS . Long-term differences in language and cognitive function after childhood exposure to anesthesia. Pediatrics. (2012) 130:e476–85. 10.1542/peds.2011-3822 22908104

[21] Rice D Barone S Jr. Critical periods of vulnerability for the developing nervous system: evidence from humans and animal models. Environ Health Perspect. (2000) 108:511–33. 10.1289/ehp.00108s3511 10852851 PMC1637807

[22] FDA Drug Safety Communication. FDA Review Results in New Warnings About Using General Anesthetics and Sedation Drugs in Young Children and Pregnant Women [EB/OL].

